# Deformation of an Encapsulated Leukemia HL60 Cell through Sudden Contractions of a Microfluidic Channel

**DOI:** 10.3390/mi12040355

**Published:** 2021-03-25

**Authors:** Mohammad Nooranidoost, Ranganathan Kumar

**Affiliations:** Department of Mechanical and Aerospace Engineering, University of Central Florida, Orlando, FL 32816, USA; mnooranidoost@knights.ucf.edu

**Keywords:** cell deformation, microfluidics, front tracking method

## Abstract

Migration of an encapsulated leukemia HL60 cell through sudden contractions in a capillary tube is investigated. An HL60 cell is initially encapsulated in a viscoelastic shell fluid. As the cell-laden droplet moves through the sudden contraction, shear stresses are experienced around the cell. These stresses along with the interfacial force and geometrical effects cause mechanical deformation which may result in cell death. A parametric study is done to investigate the effects of shell fluid relaxation time, encapsulating droplet size and contraction geometries on cell mechanical deformation. It is found that a large encapsulating droplet with a high relaxation time will undergo low cell mechanical deformation. In addition, the deformation is enhanced for capillary tubes with narrow and long contraction. This study can be useful to characterize cell deformation in constricted microcapillaries and to improve cell viability in bio-microfluidics.

## 1. Introduction

Microfluidics has emerged as a very stable and robust platform for droplet-based bio-microfludic systems and biomechanical applications such as cell deposition [1], cell encapsulation [2,3,4] and cell sorting [5,6]. Droplet-based microfluidics facilitates the production of cell-laden droplets, which eases the process of cell banking [7], cancer therapy [8] and migration of cells in in vitro culture conditions. The production of cell-laden droplets provides a functional platform for single-cell studies. In such systems, rheological shell fluids encapsulate the cells and protect them from potential exposure and the external environment. The shell provides an environment similar to that experienced in vivo [9,10], where the cell can stay viable.

Researchers have performed several experiments and used different methods to measure localized or whole-cell mechanical deformation under different conditions [11]. These techniques are adapted and modified to model the mechanics of deformation for different types of biological cells and subcellular components such as the cytoskeleton and cell membrane [12,13]. They have utilized the deformation behavior and other mechanical properties of cancer cells to classify them [8,14,15,16]. Healthy and unhealthy cells can be sorted based on their deformation characteristics, which is a very useful tool for cancer therapies [8]. Experiments of Mizoue et al. [17] showed various patterns of deformation for different cell types under precise pressure control. Kaminaga et al. [18] employed deformability of cancer cells to achieve uniform distribution of cells in a micropillar array. They proposed a new technique that can be used in cellular research at the micro-scale to analyze the cell motions in microchambers. Tsai et al. [19] developed a microfluidic chip to evaluate red blood cell deformation for a wide range of cell size, shape and orientation. They provided a correlation between transit velocity and amount of deformation which can be used as a disease checker in biomedicine.

In bio-microfluidics, the geometry of the microchannel and the presence of different obstacles within the microcapillary may affect the flow pattern and change cell mechanical characteristics. These geometrical constraints enhance the shear stresses around the cell and cause cell deformation, which may lead to cell death. Thus, understanding the effects of microchannel geometry on the deformation of cell-laden droplets is of interest and provides researchers an insight into the physics of cell migration through microcapillaries and in the design of an appropriate microfluidic device for single-cell studies. Bento et al. [20] measured the deformation of red blood cells flowing through hyperbolic, smooth, and sudden-contraction microchannels. Their results show that red blood cells flowing through a hyperbolic contraction experience a strong extensional flow with a region of homogeneous strain rate along the centerline. Therefore, the high deformability of red blood cells within the hyperbolic-shaped microchannels makes these channels a promising tool to perform sensitive cell deformability measurements for clinical purposes and early detection and diagnosis of blood diseases.

Rheology of the fluid is another important property of the fluid in characterizing flow behavior, especially in biological flows where fluids exhibit non-linear viscoelastic behavior. In these types of viscoelastic fluids, the mechanical stress has a non-linear relationship with the strain rate. The large polymeric molecules in viscoelastic fluids often show elastic behavior due to the stretching and coiling of the polymeric chains, which enhance the complexity of the flow and significantly enrich the flow behavior [21]. Polymeric concentration and relaxation time of the fluid in microfluidic systems affect the flow dynamics which can be adjusted for specific desired applications. Altering these rheological properties in bio-microfluidic systems will manipulate the cell shape deformation, which may lead to cell death [22]. The focus of this study is to numerically study the migration of encapsulated HL60 cells through sudden contractions of an axisymmetric microcapillary. In our previous works we investigated the effects of shell fluid polymeric concentration for different types of leukemia cells [23]. Following our previous work, a parametric study is done to investigate the influence of shell fluid relaxation time, as well as contraction geometry and size of the cell-laden droplet on the flow dynamics inside the channel and the cell mechanical deformation. The two main geometrical parameters that can characterize the flow dynamics and cell deformation are: (1) constriction narrowness, and (2) constriction length, whose effects on cell deformation are explained in detail throughout the paper. Other mechanical properties such as encapsulating droplet size and relaxation time are also investigated to reduce cell deformation. The simulations performed in this paper for various relevant parameters will be useful in designing a proper microfluidic device. In addition, the information provided in this paper can guide biomedical researchers to improve cell viability in the bio-microfluidic systems.

## 2. Problem Statement

**Figure 1:**
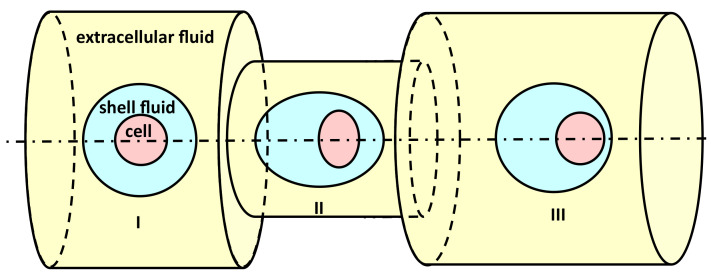
The schematic illustration of cell-laden droplet passing through a constricted microcapillary.

The migration of an encapsulated HL60 cell through sudden contraction of a microfluidic channel is shown schematically in Figure 1. The axisymmetric constricted microfluidic geometry has a circular cross-section, with diameter (D=80 μm), and a cylindrical contraction in the middle of the channel with a diameter smaller than that of the main microchannel. The cell, the shell fluid, and the extracellular fluid are shown in yellow, blue, and red colors, respectively (Figure 1). Both cell and encapsulating droplets initially have spherical shapes, and make a concentric compound droplet. They move with the flow that is initiated by imposing a fully developed velocity profile at the inlet, with an average velocity of 0.01 m/s.

The HL60 cell is shown to be more viable with less deformability compared to other leukemia cell types such as Neutrophil and Jurkat [23]. Thus, the HL60 cell simulations are more robust and stable, and the cell deformation can be calculated more precisely and accurately. This cell type is chosen in this paper for its computational stability, deformation accuracy and computational cost. The HL60 cell and the shell fluid are modeled as viscoelastic fluids, and their viscoelastic characteristics are represented by a FENE-CR viscoelastic model (Finite Extendable Nonlinear Elastic-Chilcott and Rallison) [24]. The FENE-CR model helps capture the relaxation mechanism and finite extensibility of protein-based components of the cell and the polymeric shell fluid. In this model, total viscosity consists of two components: solvent viscosity and polymeric viscosity. The polymeric viscosity ratio is defined as the ratio of polymeric viscosity to the total viscosity. The HL60 cell has a total viscosity of 40 cP, polymeric viscosity ratio of 0.7, the relaxation time of 0.17 s, and density of 1.083 kg/m${}^{3}$[1,25,26]. The cortical tension of HL60 cell is equal to 155 pN/[27]. Our polymeric shell fluid has a total viscosity of 2 cP, polymeric viscosity ratio of 0.5, and density of 10${}^{3}$ kg/m${}^{3}$[23]. The shell fluid relaxation time is adjusted to investigate its effect on cell mechanics. An FC-40 oil, with viscosity of 4 cP and density of 1.85 3 kg/m${}^{3}$, is used as the extracellular fluid, due to its biocompatibility and accessibility [28]. The interfacial tension coefficient of the shell fluid / FC-40 oil interface is set to be $\sigma_{o}=52$ mN/m [28].

## 3. Numerical Method

The flow governing equations are expressed in the framework of a one-field formulation throughout the computational domain, including: the cell, the shell fluid, and the extracellular fluid. Thus, the incompressible Navier Stokes equations can be written as:

(1) $$\begin{array}{ccc} \nabla \cdot u & = & 0, \end{array}$$

(2) $$\begin{array}{ccc} \frac{\partial \rho u}{\partial t}+\nabla \cdot \left(\rho \mathrm{uu}\right) & = & -\nabla p+\nabla \cdot \mu_{s}\left(\nabla u+\nabla u^{T}\right)+\nabla \cdot \tau +\int_{A}\sigma \kappa n\delta \left(x-x_{f}\right)dA, \end{array}$$

where ρ, $\mu_{s}$, *p*, **u**, and τ denote the density, solvent viscosity, pressure, velocity vector, and the viscoelastic extra stress tensor. The last term on the right side of Equation (2) represents the interfacial tension, where σ, κ, n, δ, and $x_{f}$ are interfacial tension coefficient, mean curvature, outward unit vector normal to the interface, and three-dimensional delta function, respectively. The viscoelastic term (∇.τ) is incorporated into the Equation (2) to characterize the viscoelasticity of the cell and the polymeric shell fluid. A FENE-CR model [24] is used to calculate the viscoelastic extra stress tensor (τ). Thus, the viscoelastic constitutive equation is expressed as:

(3) $$\begin{array}{ccc} \frac{\partial A}{\partial t}+\nabla \cdot \left(uA\right)-{\left(\nabla u\right)}^{T}\cdot A-A\cdot \nabla u & = & -\frac{L^{2}}{L^{2}-\mathrm{trace}\left(A\right)}\frac{A-I}{\lambda }, \end{array}$$

(4) $$\begin{array}{c} \tau =\mu_{p}\left(\frac{L^{2}}{L^{2}-\mathrm{trace}\left(A\right)}\right)\frac{A-I}{\lambda }, \end{array}$$

where **A**, λ, *L*, **I**, and $\mu_{p}$ are the conformation tensor, the relaxation time, the extensibility parameter, the identity tensor, and polymeric viscosity, respectively. Following Izbassarov and Muradoglu [29], the extensibility parameter for the cell and the encapsulating droplet fluid is assumed to be the same and specified as L=15. In this model the conformation tensor (**A**) represents the deformation of polymer molecules within the viscoelastic fluids in different directions, and is used to calculate the viscoelastic extra tensor (τ).

A front-tracking method [30] is used to track the interface between fluids. In this method, the flow field is solved on a staggered Eulerian grid, and the interfaces between different phases are resolved by a Lagrangian grid. The Lagrangian grid consists of marker points connected with front elements, and moves with the projected velocity field [29,30,31]. The interfacial tension first is calculated on the grid points of the interface (Lagrangian grid) and then is projected on the flow-field computational grids (Eulerian grids). A central difference scheme and a first-order explicit method are utilized to approximate the spatial and time derivatives.

A Fortran code is used to solve the governing Equations (1) and (2). Microchannel walls are treated with no-slip boundary conditions, and a symmetry boundary condition is used at the centerline. Constant pressure is set as the boundary condition at the outlet. A uniform Cartesian grid is employed, and a grid convergence study is done to determine the minimum grid size required to reduce the spatial discretization error below a threshold value. It is found that a computational grid containing 128 cells in the radial direction and 640 cells in the axial direction are sufficient to reduce the spatial error for all flow configurations. Therefore, this mesh is used in all the results presented in this paper.

**Figure 2:**
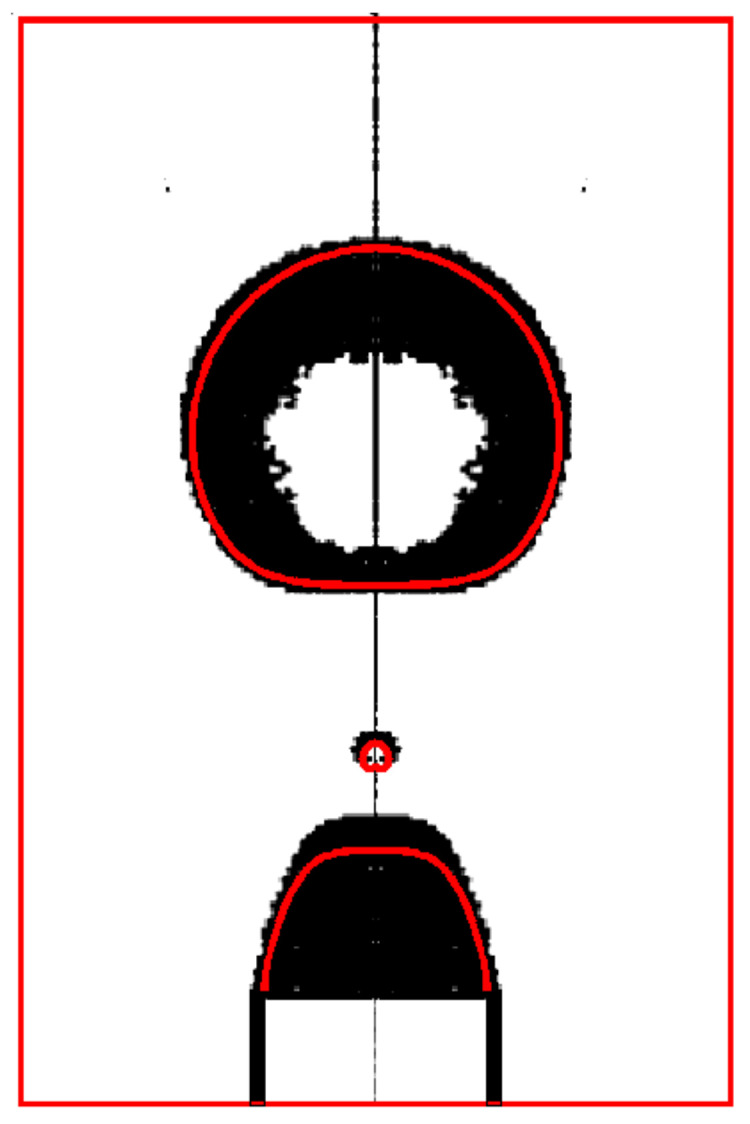
Comparison of present study (**in red**) with respect to the experiments (**in black**) of Zhang [32].

The numerical method is validated with experiments of Zhang [32] on drop formation in viscous flows. For this purpose, the formation of 2-ethyl-1-hexanol (2EH) drops in distilled water from a tube of radius R=0.16 cm is simulated for a liquid flow rate of Q=5 mL/min. The simulation is compared with the experiment [32] after the pinch-off (Figure 2). The result in the current study and the experiments of Zhang [32] are shown in red and black, and the comparison is seen to be favorable.

## 4. Results and Discussion

The migration of an encapsulated cell can be described in three different stages: (I) before contraction, (II) within the contraction, and (III) after the contraction (Figure 1). The cell-laden droplet is initially in a spherical shape. As the cell-laden droplet moves with the flow, the change in the shape of encapsulating droplet and the cell starts when it comes close to the contraction, just before the contraction entrance. Both encapsulating droplets and the cell undergo a large deformation as they move further within the contraction, and almost regain their shape downstream the channel.

**Figure 3:**
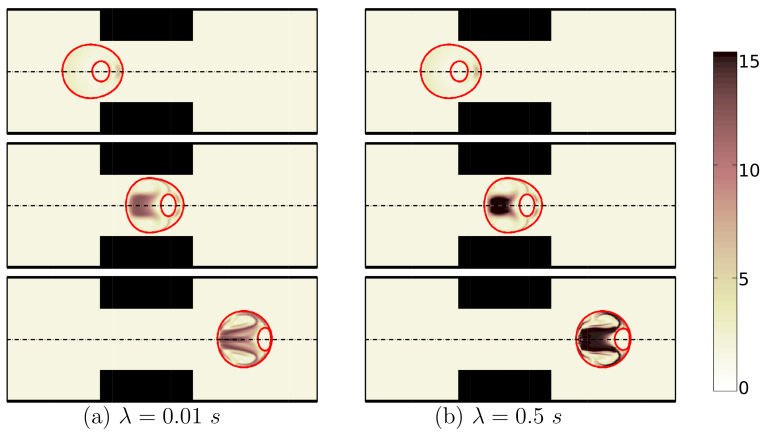
Shape evolution of an encapsulated HL60 cell through the constricted microchannel at different locations of channel. The shell fluid relaxation time is: λ=0.01 (**a**) and λ=0.5s (**b**). The polymeric viscosity ratio is fixed at β=0.5. The color bar and contours represent the square root of trace of conformation tensor inside the cell and the shell fluid (Ca=0.00076 and Re=0.37).

Relaxation time, defined as the time taken by the polymers in the viscoelastic fluid to return from the deformed state to its initial equilibrium state, is an important property of viscoelastic fluids which can influence the flow structure in bio-microfluidics. The fluids with high relaxation times get relaxed and returned to their initial state at a longer pace. Detailed simulations are performed to investigate the effects of shell fluid’s relaxation time on the deformation of an encapsulated HL60 cell and the dynamics of the encapsulating droplet. Shape evolution of both encapsulating droplet and the HL60 cell at different locations of the microchannel are shown for two different relaxation times λ=0.01 s (left half of Figure 3) and 0.5 s (right half of Figure 3). As the encapsulating shell droplet enters the contraction area, viscoelastic stresses built in the encapsulating droplet become more pronounced for a high relaxation time of λ=0.5 s. These stresses along with wall effects characterize the deformation of the cell. The viscoelastic stresses around the cell have the ability to dampen the deformation and therefore reduce the cell deformation.

**Figure 4:**
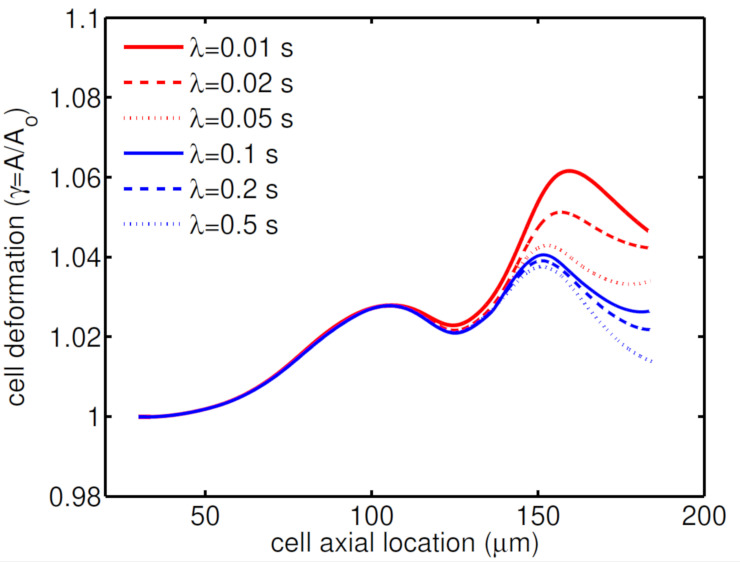
Deformation of an HL60 cell at different relaxation times for β=0.5, Ca=0.00076 and Re=0.37.

The deformation of the cell membrane is quantified by the relative cell membrane area ($\gamma =A/A_{0}$), where *A* and $A_{0}$ are the surface areas of the deformed cell and the initial spherical cell, respectively. This value is always equal or above unity. As shown in Figure 4, deformation of the HL60 cell has a bimodal behavior for all relaxation times. The first peak in the cell deformation is almost similar for all relaxation times; however, increasing relaxation time reduces the second peak in cell deformation. The minimum deformation is observed for the highest shell fluid relaxation time (λ=0.5 s).

The deformation of the cell membrane may threaten cell viability and lead to cell death [22,23]. Takamatsu and Rubinsky [33] proposed a theoretical model to calculate viability of deformed cells under mechanical stresses. They statistically computed the percentage of impaired cells for a set of experiments on cell compression between two parallel plates and derived a correlation between relative change in cell membrane area and the percentage of impaired cells. Their results show that the cell remains intact for an increase in cell membrane area up to 5%, while further increase in the cell surface area can result in a rupture and consequently death of the cell. This analytical model is used in our current study to compute probability of cell survival (η) as a function of maximum instantaneous value of cell deformation $\left(\gamma_{max}\right)$:

(5) $$\begin{array}{c} \eta \left(\gamma \right)=\left\{\begin{array}{cc} 1 & \mathrm{for}\;\;\gamma_{\max}<\gamma_{cr}-\Delta \gamma ,\\ \frac{1}{2}-\frac{\gamma_{\max}-\gamma_{cr}}{2\Delta \gamma } & \mathrm{for}\;\;\gamma_{cr}-\Delta \gamma \leq \gamma_{\max}\leq \gamma_{cr}+\Delta \gamma ,\\ 0 & \mathrm{for}\;\;\gamma_{\max}>\gamma_{cr}+\Delta \gamma , \end{array}\right. \end{array}$$

where $\gamma_{cr}=1.5$ and Δγ=0.5 are the critical cell deformation and the range of surface expansion, respectively [1,33]. The HL60 is 94% viable in the presence of shell fluid with low relaxation time (λ = 0.01 s), while its viability increase to 96% for a higher relaxation time (λ = 0.5 s).

**Figure 5:**
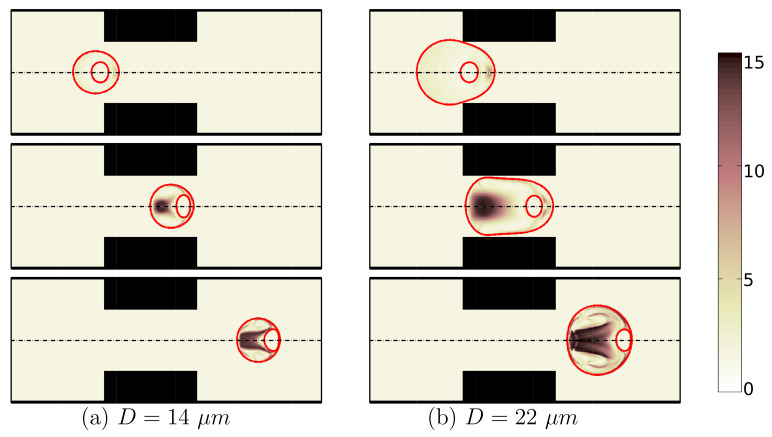
Shape evolution of an encapsulated HL60 cell through the constricted microchannel at different locations of channel. The encapsulating droplet radius is: D=14 μm (**a**) and D=22 μm (**b**). The color bar and contours represent the square root of trace of conformation tensor inside the cell and the shell fluid. (λ=0.1s, β=0.5, Ca=0.00076 and Re=0.37).

**Figure 6:**
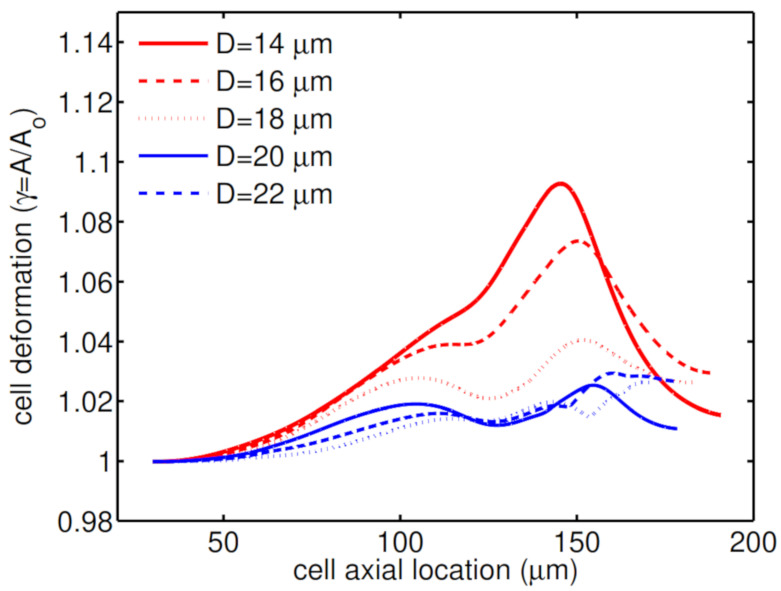
Deformation of an HL60 cell for different radii of encapsulating droplet at λ=0.1s, β=0.5, Ca=0.00076 and Re=0.37.

Further simulations are performed for encapsulating droplets with different radii. As shown in Figure 5, a large compound droplet with radius (D=22 μm) does not experience a severe constraint and therefore remains almost spherical; while a small compound droplet with radius (D=14 μm) elongates mainly due to the geometrical constraint as it passes through the constriction. Figure 6 shows how the compound droplet size affects the cell deformation.

**Figure 7:**
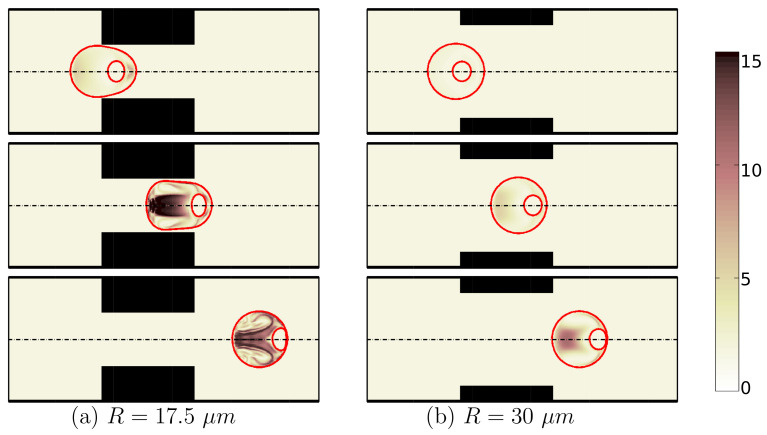
Shape evolution of an encapsulated HL60 cell through constricted microchannels with R=17.5 μm (**a**) and R=30 μm (**b**) at different locations of channel. The color bar and contours represent the square root of trace of conformation tensor inside the cell and the shell fluid. (λ=0.1 s, β=0.5, Ca=0.00076 and Re=0.37).

The constriction in the middle of the channel dictates the flow structure and can significantly change the shape of the encapsulating droplet and deformation of the cell. This constriction restricts encapsulating droplet movement as it migrates downstream the channel. The two important geometrical properties which characterize the constricted microchannel are the length of the constriction (*L*) and the narrowness of the cross-section in the constriction area (*R*). The effects of these two properties on deformation are studied for an encapsulated HL60 cell. Figure 7 shows that the encapsulating droplet experiences high viscoelastic stresses and undergoes a high deformation with the viability of 91% in the presence of a narrow constriction in the middle of the channel (R=17.5 μm). However, for a relatively wider constriction (R=30 μm), the encapsulating droplet experiences low viscoelastic stresses and almost remains spherical with the viability of 98% as it passes through the constricted microcapillary.

**Figure 8:**
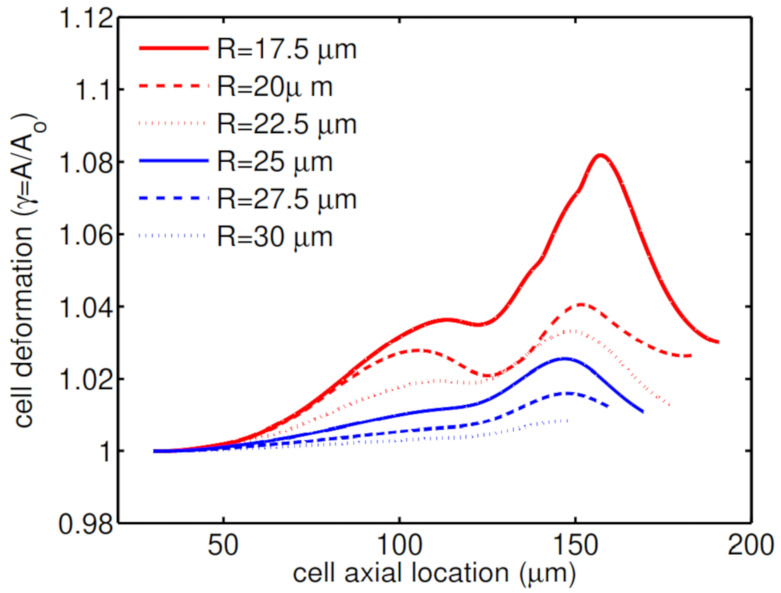
Deformation of an HL60 cell passing through microchannels with different constriction narrowness at λ=0.1 s, β=0.5, Ca=0.00076 and Re=0.37.

Figure 8 shows how cell deformation rate is different for microcapillaries with the different narrowness of the constriction. As the constriction gets wider, the encapsulating droplet can move more freely in the channel which helps in having a low cell deformation and high viability of 98%.

**Figure 9:**
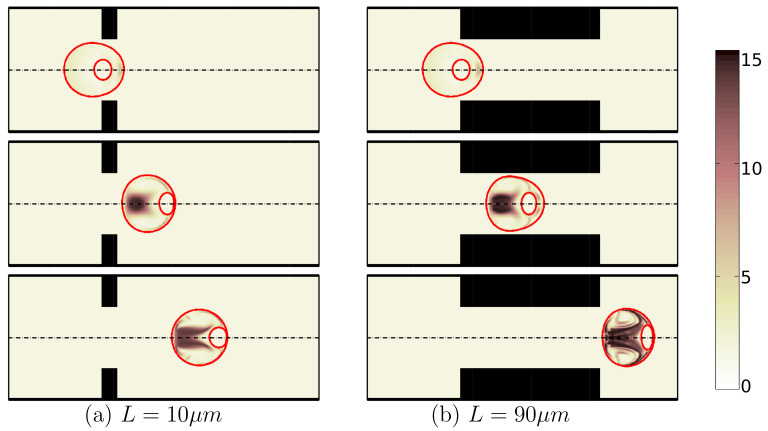
Shape evolution of an encapsulated HL60 cell through constricted microchannels with L=10 μm (**a**) and L=90 μm (**b**) at different locations of channel. The color bar and contours represent the square root of trace of conformation tensor inside the cell and the shell fluid. (λ=0.1 s, β=0.5, Ca=0.00076 and Re=0.37).

**Figure 10:**
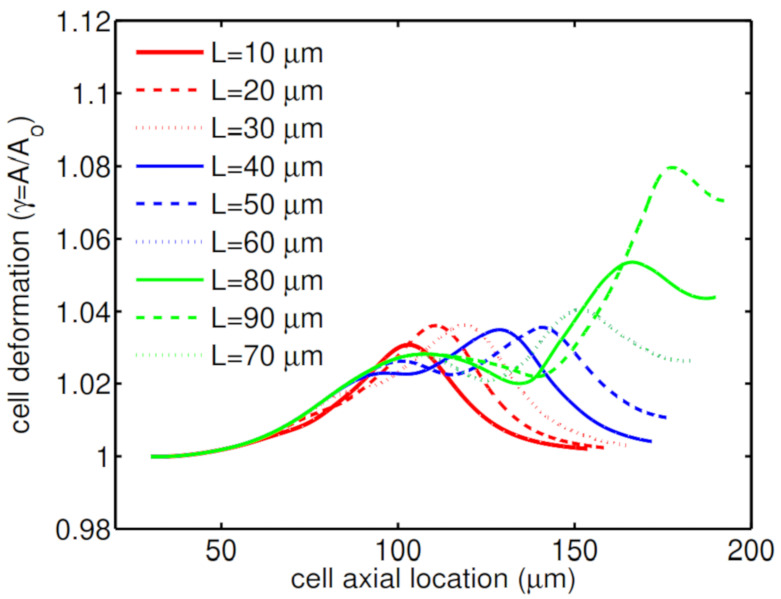
Deformation of an HL60 cell passing through microchannels with different constriction lengths at λ=0.1 s, β=0.5, Ca=0.00076 and Re=0.37.

Another geometry characteristic that has a big influence on cell deformation is the constriction length. Simulations show that for a short constriction, the cell is under shear stress for a short period of time and therefore this small shear stress cannot threaten its life. As a result, the cell undergoes a low mechanical deformation (Figure 9a), which results in high cell viability of 99%. On the other hand, for a microchannel with a long constriction in the middle of channel, the cell is under shear stress for a longer time, which increases the cell deformation through its passage along the channel (Figure 9b). Figure 10 shows how the second peak in the cell deformation is larger in the case of a long constriction within the microchannel, which reduces the cell viability to 92%.

## 5. Conclusions

A parametric study has been performed to investigate the influence of shell fluid relaxation time, the encapsulating droplet size and contraction geometry on the deformation of an encapsulated HL60 cell passing through sudden contractions within a microcapillary. A three-phase front-tracking method is used to simulate the flow dynamics of encapsulated cells in the microchannel and to investigate the deformation of an encapsulated HL60 cell during its passage through the contractions of a microcapillary.

Shell fluid rheology is found to help reduce cell deformation. In particular, in the case of an HL60 cell covered with a high polymeric shell fluid, the cell undergoes low mechanical stresses which help it remain its spherical shape. Membrane area of an HL60 cell covered with a low relaxation time shell fluid (λ=0.001 s) is changed 6% at maximum; however, a high polymeric shell fluid (λ=0.5 s) reduces the cell membrane area relative change to 4%. The cell membrane area doesn’t change by further increasing the relaxation time. The cell deformation decreases as the encapsulating droplet enlarges. A small cell-laden droplet D=14 μm experience a maximum 10% change in the cell membrane area during the cell-laden droplet through the constriction. This change in cell deformation is decreased to 2% in the case of a relatively large droplet D=22 μm.

The effects of two main geometrical characteristics including the constriction narrowness and length were also studied for an HL60 cell encapsulated by a viscoelastic shell fluid. The simulations suggested that short constrictions cannot have a significant influence on cell mechanical properties and the cell remains almost spherical. In the case of a long constriction, the cell experiences high shear stresses for a long time which affects its membrane shape. The narrow constriction also significantly impact cell dynamics, which threatens the cell viability.

In other words, to have a minimum cell deformation, the constriction size needs to be short and wide enough for the cell to freely pass through the microchannel. A small encapsulating droplet with high relaxation time helps reduce the deformation rate.

## Data Availability

Not applicable.
